# Evaluating the effect of a 12-month youth advisory group on adolescent’s leadership skills and perceptions related to chronic disease prevention research: a mixed-methods study

**DOI:** 10.1186/s12889-023-17283-2

**Published:** 2023-11-27

**Authors:** Mariam Mandoh, Rebecca Raeside, Allyson Todd, Julie Redfern, Seema Mihrshahi, Hoi Lun Cheng, Philayrath Phongsavan, Stephanie R Partridge

**Affiliations:** 1 School of Health Sciences, Faculty of Medicine and Health, The University of Sydney, Camperdown, NSW 2006 Australia; 2 The George Institute for Global Health, The University of New South Wales, Camperdown, NSW 2006 Australia; 3 Charles Perkins Centre, The University of Sydney, Sydney, NSW 2006 Australia; 4 Department of Health Sciences, Faculty of Medicine Health and Human Sciences, Macquarie University, Sydney, NSW 2109 Australia; 5 Sydney Medical School, Faculty of Medicine and Health, Specialty of Child and Adolescent Health, The University of Sydney, Westmead, NSW 2145 Australia; 6 Academic Department of Adolescent Medicine, The Children’s Hospital at Westmead, Westmead, NSW 2145 Australia; 7 Prevention Research Collaboration, Sydney School of Public Health, Faculty of Medicine and Health, The University of Sydney, Camperdown, NSW 2006 Australia

**Keywords:** Youth advisory, Adolescents, Participation, Engagement, Action research, Chronic Disease, Prevention, Youth, Young people

## Abstract

**Background:**

Youth Advisory Groups (YAGs) represent a promising method to engage adolescents in research of relevance to them and their peers. However, YAGs are rarely implemented or evaluated in chronic disease prevention research. The aims of this study were firstly, to evaluate the effect of participation in a 12-month YAG on adolescents’ leadership skills and perceptions related to chronic disease prevention research and secondly, to evaluate the process of establishing and facilitating a 12-month YAG and identify barriers and enablers to establishment and facilitation.

**Methods:**

This study was a 12-month pre-post study. Eligible participants were adolescents (13-18-years) and current members of an established YAG. Data collection involved online surveys and semi-structured interviews at baseline, six-months and 12-months follow-up. Participatory outcomes such as self-efficacy, leadership skills, and collective participation were derived from Youth Participatory Action Research Principles (YPAR), and the Lansdown-UNICEF conceptual framework for measuring outcomes of adolescent participation. Process evaluation data were captured via meeting minutes, Slack metrics and researcher logs. Quantitative data was analysed using descriptive statistics and qualitative data was thematically analysed using a reflexive thematic analysis approach.

**Results:**

Thirteen (13/16) YAG youth advisors consented to participate in the evaluation study (mean age 16.0 years, SD 1.3; 62% (8/13) identified as female). Survey data assessing participatory outcomes found an increase in leadership and life skills scores over 12-months (+ 8.90 points). Semi-structured interview data collected over the 12-month term revealed three key themes namely: influence, empowerment, and contribution. Comparison of pre-post themes determined a positive trend at follow-ups, demonstrating improved participatory outcomes. Process indicators revealed that at 12-month follow-up the YAG was implemented as planned. Semi-structured interview data determined barriers to YAG facilitation included time and limited face-to-face components, while enablers to YAG facilitation included flexibility, accessible delivery methods, and a supportive adult facilitator.

**Conclusion:**

This study found that a YAG fostered positive participatory outcomes and unique opportunities for youth participants. A successful YAG based on YPAR principles requires researchers to ensure YAG establishment and facilitation is an iterative process. Taking into consideration important barriers and enablers to YAG facilitation ensures adolescent engagement in a YAG is both meaningful and impactful.

## Background

In society today, adolescents (10-19-years) [1] face unique and evolving challenges [2]. The combination of challenges includes, but are not limited to, the rapidly changing digital landscape, the climate crisis, inequitable food systems, economic uncertainty, and the ongoing implications of the Coronavirus Disease 2019 (COVID-19) pandemic [3–8]. These societal issues present proximal challenges for adolescents that hamper their ability to eat well and be physically active, [2] including easy access to digital environments with limited regulations and protections, low availability or affordability of healthful foods and limited options for active travel in many countries [4, 9, 10]. Unseen in previous generations, this amalgamation of challenges is not conducive to optimising health and wellbeing and subsequently adolescents, globally, are at an increased risk of developing chronic diseases earlier in adulthood [11, 12]. As researchers, we need to see these challenges through the eyes of adolescents and work with them to develop solutions [3, 10, 13].

Youth engagement in chronic disease prevention research has the potential to yield solutions to overcome such adolescent health challenges [3, 8, 14–17]. Over the past three decades the concept of youth engagement has become widely accepted [16]. Youth engagement is grounded in the 1989 United Nations Convention on the Rights of a Child (UNCRC) [18], which clearly articulates the rights of young people (< 18 years) to be involved in matters of importance to them. Notably, the significance of youth engagement extends beyond basic rights, with the World Health Organisation (WHO)-UNICEF-Lancet commission [19, 20], the 2018 World Youth Report [21], and the international, multiorganization ‘make adolescent well-being a priority: call to action’ [22] identifying adolescent engagement in decision-making in health related matters as a necessity to empower adolescents to be the change makers of the future [23], improve adolescent well-being and as a criteria to achieve health related Sustainable Development Goal (SDG) targets [23, 24].

At the end of the last decade, adolescents believed their voices in the chronic disease dialogue had been tokenised and were generally dismissed [23, 25, 26]. More recently, the 2020 UNICEF-Lancet-Financial Times commission U-report poll revealed more than 23,000 youth globally are interested in engaging in new health solutions research and call for involvement in “all stages of digital health research design, implementation and governance” [27, 28]. Such engagement in research may be realized by employing Youth Participatory Action Research (YPAR) principles, which involves adolescents constructing knowledge by identifying, researching, and addressing health problems through adolescent–adult partnerships [29]. A recent systematic review of 63 adolescent-related studies employing YPAR principles, described evidence for improvements in adolescents’ skill development [30]. However, only 19 studies were related to health, and adolescents were predominantly research participants with a passive role as opposed to research collaborators [30]. Further, a systematic scoping review investigating the mode and nature of adolescent participation in chronic disease prevention research [31, 32] identified only six studies which engaged adolescents in an adolescent-led capacity where they had influence over the entire process and outcomes of the research, while adults operated as facilitators. Of the adolescent-led studies, youth advisory groups, youth advocacy and peer leadership were shared components. Despite these studies recognising the importance of adolescent participation, participatory outcomes were rarely measured, and these studies rarely evaluated [32].

Initiatives that involve active youth participation throughout the research cycle reported improved participatory outcomes [31, 32]. Participation in a formal youth advisory capacity (e.g., Council, Board, Group) involves adolescents working in partnership with researchers at various phases of the research cycle. Evaluation of a twelve-month international youth council involving sixteen young people aged 13–23 years old found that members reported that participation had a positive impact on the transition from paediatric to adult care for youth with a chronic condition [33]. In a global context a health focused Youth Advisory Group (YAG) during the COVID-19 pandemic empowered adolescents to find solutions to adolescent health needs during the pandemic [34, 35]. Nonetheless, in 2019, researchers found that less than 1% of all empirical child and adolescent studies published that year used a YAG to inform their child or adolescent focused health research [36, 37].

The use of YAGs epitomises an innovative [33] and practical strategy to meaningfully engage adolescents in an adolescent-led approach throughout all phases of the research cycle [38, 39] to enhance research development and translation [34, 36, 40]. However, the lack of YAG evaluation studies and measurement of participatory outcomes leaves a gap in the adolescent participation in chronic disease prevention research literature. The aims of this study were to, firstly, evaluate the effect of participation in a YAG on adolescents’ leadership skills and perceptions related to research and chronic disease prevention and secondly, to evaluate the process of establishing and accommodating a 12-month YAG focused on chronic disease prevention research and to identify barriers and enablers to establishment and facilitation.

### Health Advisory Panel for Youth at the University of Sydney (HAPYUS) YAG structure

The HAPYUS YAG was designed as a leadership initiative to allow adolescents to collaborate with a team of four full-time public health researchers to identify and advocate for research issues that matter to them. Adolescents were given the opportunity to contribute towards and help shape chronic disease prevention research projects [3, 41, 42]. This opportunity aimed to help create leadership and capacity building opportunities for adolescents within the research sector. Participation in the YAG aimed to help increase adolescents’ self-efficacy, leadership skills and research aptitude through skill development sessions with researchers and health professionals in chronic disease prevention field. The YAG consisted of three co-chairs and 13 general members aged 13 to 18 years. Co-chairs were responsible for taking the lead on activities led by the YAG and ensured that communication and engagement on the online platform was safe, inclusive and respectful. Current evidence on research- and health-related YAGs suggests YAG of 16 members is manageable and ensures members are engaged throughout their 12-month term [36]. Sixteen members was also an appropriate size for our research team’s capacity and composition [43]. Members were recruited via an application process from across NSW, Australia via paid advertising on social media and were asked demographic questions and asked to respond two questions (maximum 150 words for each) ‘Why do you want to join our Youth Advisory Group? Please don’t feel pressured to write a lot. We just want to know a bit more about you and why you are interested in being involved)’ and ‘What life experiences have you had which would assist you in contributing to the Youth Advisory Group? (e.g., volunteering, leadership, community engagement?)’ The research team selected 16 members based on their responses to the two questions and demographic data to ensure the group was gender balanced, with representation of adolescents from different ethnic backgrounds and regional and rural areas. All members were reimbursed with AUD$30 gift voucher for their time each month. The YAG was responsible for collaborating with the research team on research relating to adolescents, chronic disease prevention and other associated research; identifying opportunities for development of key skills for adolescents related to research; advocating for adolescents to be engaged in research for the prevention of chronic diseases; developing and monitoring strategies and initiatives to engage adolescents in research; and ensuring broad and inclusive representation of adolescents views especially those without direct group representation. YAG members were given the opportunity to decide on a name for the group, upon consensus the YAG was named the Health Advisory Panel for Youth at the University of Sydney (HAPYUS).

### Framework for collaboration

The HAPYUS YAG was informed by Youth Participatory Action Research (YPAR) principles [44, 45] and by the guidelines on adolescent participation and civic engagement, specifically the essential features of meaningful adolescent participation [46, 47] and theory of change for adolescent participation [48]. The 12-month term required youth advisors to participate in monthly 90-minute video teleconferencing youth-led meetings that consist of a 30-minute research skill development session led by the research team, guest researcher or health professional. The research skill development sessions were iterative, and topics were selected by members of the research team over the course of the YAG. Research skill topics included, but were not limited to YPAR principles, cooperative learning, and teamwork; conducting effective, beneficial, and useful research projects; advocacy; and consent and assent. Following the research skill development sessions was a 60-minute youth advisory meeting guided by the YPAR principles: (i) inquiry based, topics of discussion were grounded in youth advisors lived experience and concerns related to chronic disease prevention; (ii) participatory, youth advisors are collaborators in the research process; and (iii) transformative, youth advisors actively intervene to change research to improve the lives of youth and their communities from the negative impacts of chronic diseases. The essential features of meaningful participation state that ‘adolescents need safe and inclusive opportunities that provide them with space and time to form and freely express their views and opinions’; adolescents should be provided appropriate information to inform their views, and they should be able to use the media of their choice to communicate their views and to negotiate decisions’; ‘adolescents’ views must be respectfully and seriously heard by those with the power and authority to act on them’; and ‘adolescents’ views should receive proper consideration, and adolescents should receive timely feedback about the outcome(s) and the extent of their influence.’ In-between meetings youth advisors engaged and contributed to topics of discussion and collaborated on research projects using Slack, an online communication platform. The Slack team was accessible only to the 16 youth advisors and members of the research team.

## Methods

### Study design

**Fig. 1:**
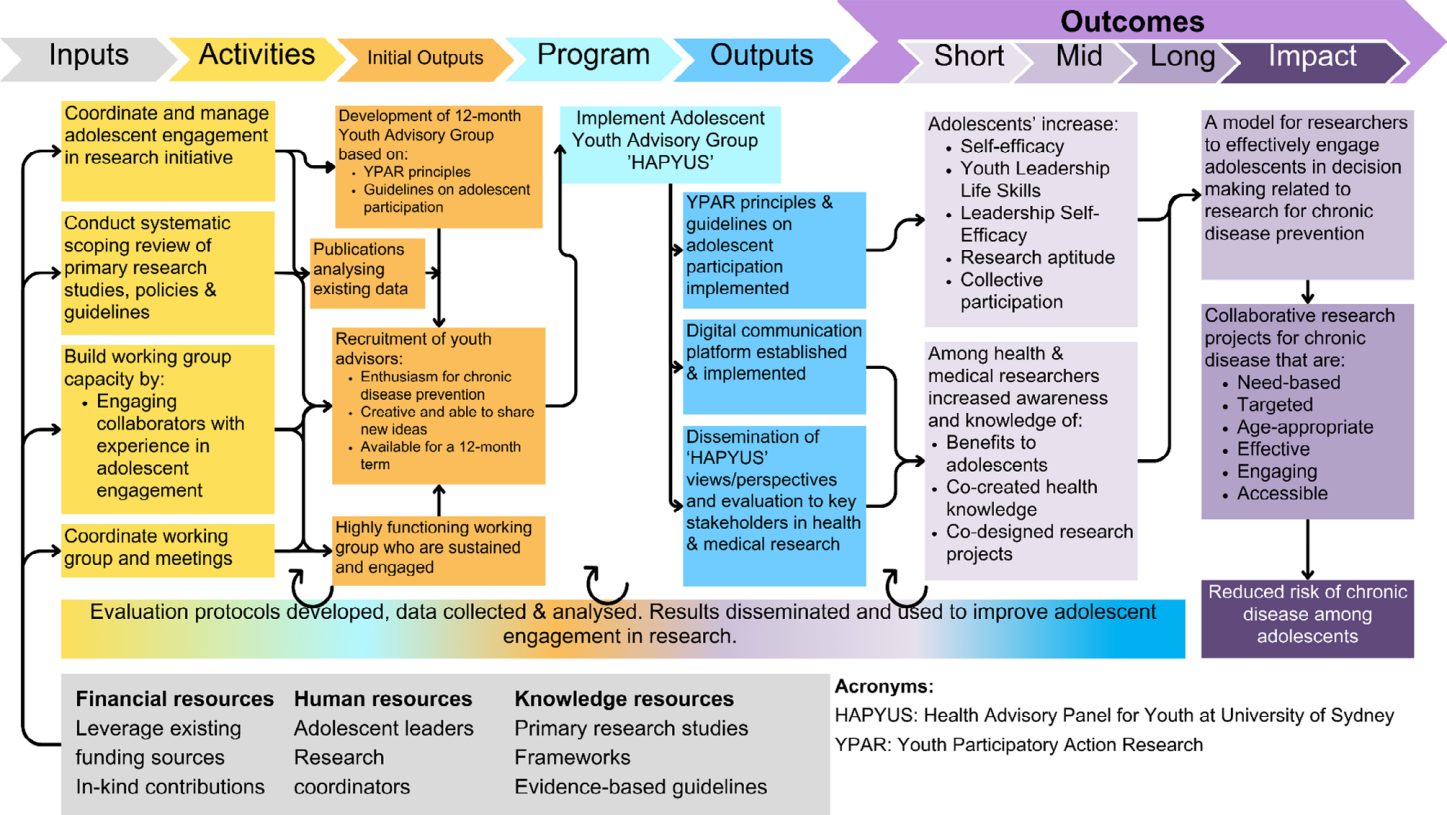
YAG Evaluation Logic Model

The Youth Advisory Group (YAG) evaluation was a 12-month convergent parallel mixed-methods pre-post study design, approved by the University of Sydney Human Research Ethics Committee (Approval No. 2021/749). Research techniques, using a variety of qualitative and quantitative methods are common components and strengths of youth participatory action research studies [29] and an appropriate methodology to assess the effects of participatory research on adolescents and their environment [49]. A logic model describing the YAG structure and evaluation is presented in Fig. 1. This study presents short term outcomes related to adolescents’ leadership skills and perceptions related to research and chronic disease prevention.

### Participants

Adolescents aged 13–18 years (inclusive), who were members of a YAG at The University of Sydney and provided informed e-consent. All youth advisors participating in the established HAPYUS YAG were invited to participate in the YAG evaluation study after the first meeting at the start of their 12-month term. The WHO recognises adolescents as young people aged between 10 and 19 years [1], however the YAG only included young people aged 13–18 years as this age range coincides with a higher level of independence and with secondary education in New South Wales, Australia where the study was conducted. Participation in the evaluation component was voluntary and a decision not to participate did not impact their youth advisory group role.

### Data collection and study outcomes

Demographic data was collected at baseline and derived from standardised Australian national surveys. Participants provided self-reported data on age (years), gender identity (male, female, non-binary/gender diverse, other or prefer not to say), postcode (for categorizing socioeconomic Indexes for Areas (SEIFA) and major city, regional or remote residential areas), school attendance and language spoken at home. Participants’ residential postcodes were used to determine the SEIFA Index of Relative Socioeconomic Disadvantage (IRSD) [50] quintile (quintile 1 representing the 20% most disadvantaged areas) and the Australian Statistical Geographical Standard Remoteness Areas (ASGS-RA) [51] as a measure of geographical location and relative access to services.

### Outcome evaluation

Short-term outcome indicators of interest are presented in Fig. 1. and include participatory outcomes derived from the Lansdown-UNICEF conceptual framework for measuring outcomes of adolescent participation [46]. The Lansdown-UNICEF framework [46] does not provide comprehensive evaluation measures, therefore the evaluation framework was adapted using established surveys which have demonstrated good reliability and validity in adolescent populations [52–56]. Table 1. describes the YAG participatory outcome indicators of interest and the chosen assessment tool. The General Self-Efficacy scale (GSE) [52] measured self-efficacy, self-confidence and ability to make changes, the Leadership/ Teamwork Self-Efficacy Scale (LTSES) [53, 54] assessed changes in leadership, teamwork, and collaboration indicators and the Youth Leadership Life Skills Development Survey (YLLSDS) [55, 56] measured youth leadership and engagement skills such as communication, decision-making, learning, confidence, problem-solving and group work in the participatory research setting (Table 1.).

**Table 1:** Description of YAG outcome evaluation indicators and assessment

Participatory Outcome indicator (Based on the Lansdown-UNICEF framework)	Assessment			
	Outcome measured	Survey	Description	Reliability and Validity
Participatory outcomes associated with empowerment and influence, including: i) **Sense of self-worth, self-esteem, or efficacy**, - characterised by self-confidence and the ability to make changes. ii) **Being taken seriously**, - connected with sense of influence, motivation, potential to make a difference and opportunities. iii) **Making decisions**, - characterised by self-confidence, improved knowledge, sense of responsibility and adults’ confidence in adolescents’ abilities; and iv) **Public or civic engagement**, - related to learning and knowledge, potential to influence issues of importance, sense of responsibility, opportunities and collaboration [45].	Self-efficacy, self-confidence, ability to make changes	General Self-Efficacy scale (GSE) [51]	The GSE is 10-item scale, with responses made on a 4-point scale ranging from ‘not at all true’=1 to ‘exactly true’=4. The total score for the ten GSE items ranges from 10 (lowest) to 40 (highest total score), where higher scores indicate higher GSE.	In data from 23 countries, GSE was found to be valid and reliable with a Cronbach’s alpha between 0.76 and 0.90 [51].
	Leadership, teamwork, and collaboration indicators	Leadership/ Teamwork Self-Efficacy Scale (LTSES) [52, 52]	The LTSES is a 10-item scale designed to measure perceived ability to collaborate, communicate with and lead others within a research team context. Items are measured on a 5-point Likert-type scale ranging from ‘strongly disagree’=1 to ‘strongly agree’=5. The total score for the ten LTSES ranges from 10 (lowest) to 50 (highest total score). There are two subscales for teamwork factors and leadership factors ranging from 5 (lowest) to 25 (highest sub score) for both subscales.	The LTSES has a good internal reliability and validity, with Cronbach’s alpha > 0.50 [52, 53].
	Youth leadership and engagement skills, such as: communication, decision-making, learning, confidence, problem-solving and group work in the participatory research setting	Youth Leadership Life Skills Development Survey (YLLSDS) [54, 55]	The YLLSDS is a 30-item survey that ask participants to select a response of ‘no gain’=0 to ‘a lot of gain’=3 when reflecting on their experience being a part of the YAG. The total score for the 30 YLLSDS items ranges from 0 (lowest) to 90 (highest total score) indicating the most gain in leadership life skills.	The YLLSDS has a high internal reliability and validity with Cronbach’s alpha > 0.90 [54].

A purpose built semi-structured interview guide was developed by the research team to encompass and build upon the scope of participatory outcomes outlined in the Lansdown-UNICEF framework [46] and the theory of change for adolescent participation [48]. The semi-structured interview questions were open-ended and broad to contextualise the nature and extent of participatory experience, research skills, confidence, experience of being listened to and taken seriously, making decisions, sense of engagement and any unexpected outcomes of involvement in participatory research activities that may emerge. Baseline semi-structured interviews were conducted by two members of the research team (MM, SRP). At baseline all youth advisors were new to the study and did not know the research staff. At six-months follow-up semi-structured interviews were conducted by one researcher (MM), who had limited involvement in the youth advisory meetings and activities. To avoid courtesy bias, 12-month semi-structured interviews were conducted by one researcher (AT), who was external to the YAG study team and unknown to the study participants.

Quantitative survey data and qualitative semi-structured interview data were captured from participants at baseline, six-months, and 12-months except the YLLSDS, which was only administered at 6-months and 12-months. Surveys were administered online via the Research Electronic Data Capture (REDCap) platform and semi-structured interviews were conducted via Zoom teleconferencing software.

### Process evaluation

To evaluate the process of establishing and accommodating a 12-month YAG and address aim two, we measured activities and outputs to indicate whether the program was implemented as planned. We documented meeting minutes to capture participant’s attendance at monthly Zoom meetings from all 16 members. Slack workspace metrics over the 12-months were downloaded and analysed, including number of active weekly members, total number of messages sent, where conversations happened, and the number of files uploaded. Further, barriers and enablers to establishing and accommodating the 12-month YAG were identified from ongoing correspondence with participants via Slack and as feedback in monthly Zoom meetings.

### Data analyses

Descriptive statistics were reported for continuous and categorical survey data. Semi-structured interviews were audio recorded on Zoom, transcribed verbatim and thematically analysed in NVIVO version 12.0 (QSR international). Braun and Clarke’s reflexive thematic analysis approach was determined to be the most suitable method of analysis of the qualitative semi-structured interview data [57, 58]. Reflexive thematic analysis is an approach involving reflexive, repetitive engagement with the dataset to generate a strong analysis [59, 60]. Braun and Clarke emphasise thematic analysis to be a recursive process, outlining six phases to guide analysis: (1) Familiarisation with the dataset; (2) Coding; (3) Generating initial themes; (4) Developing and reviewing themes; (5) Refining, defining, and naming themes; (6) Writing up. Thematic analysis was led by one researcher (MM), who coded the interviews, a second researcher (AT) crosschecked 20% of interviews for consistency. MM and AT then met to compare, discuss, and resolve any inconsistencies. Direct quotations from the semi-structured interviews were used to demonstrate consensus among participants and individual opinions regarding key themes. The shift in themes over the 12-month study period (negative, positive, neutral) were discussed between two researchers (MM and AT) until consensus was reached.

## Results

Thirteen of the 16 members of the youth advisory group provided informed consent to participate and completed baseline assessments. Eleven and ten participants completed assessments at 6-months and 12-months, respectively. The mean age of participants was 16.0 years, Standard Deviation (SD) 1.3. Eight (8/13; 62%) participants were enrolled in the final two grades of high school. Eight (8/13; 62%;) participants identified as female. Five (5/13; 38%) participants reported a language other than English as the main language spoken at home. Four (4/13; 31%) participants reported living in a rural or remote suburb. Eight (8/13; 62%) participants lived in a suburb with an IRSD score in the top two quintiles, reflecting a relative lack of disadvantage and higher socio-economic status.

### Outcome evaluation data

Table 2 presents results on the key participatory outcomes. Participants reported high mean scores on general self-efficacy, leadership, and teamwork skills scales at all timepoints with no meaningful change over the 12-month period. However, when reflecting on their experience participating in the YAG in the YLLSDS at six- and 12-months intervals, an increase in mean scores was apparent.

**Table 2:** Effect of participation in a youth advisory group on adolescents’ self-efficacy, leadership, and teamwork skills

Outcome measures	Baseline (n = 13)	6-months (n = 11)	12-months (n = 10)	Mean difference^2^ (baseline-6 months)	Mean difference^2^ (6–12 months)	Mean difference^2^ (baseline-12 months)
**GSE** total (10–40)						
Mean score (SD)	34.08 (3.77)	31.55 (3.83)	33.90 (3.54)	-2.53	+ 2.35	-0.18
Median score (mode)	34 (39)	31 (28)	35 (36)			
**LTSES** total (10–50)						
Mean score (SD)	46.23 (3.83)	44.36 (5.14)	45.50 (4.45)	-1.87	+ 1.14	-0.73
Median score (mode)	47 (50)	46 (43)	46.5 (50)			
**LTSES Leadership factors** (5–25)						
Mean score (SD)	23.08 (2.22)	22.91 (2.47)	22.90 (2.89)	-0.17	-0.01	-0.18
Median score (mode)	24 (25)	23 (25)	23 (25)			
**LTSES Teamwork factors** (5–25)						
mean score (SD)	23.15 (2.07)	21.45 (3.88)	22.60 (2.17)	-1.70	+ 1.15	-0.55
Median score (mode)	23 (23)	23 (23)	23 (24)			
**YLLSDS**^1^ (0–90)						
Mean score (SD)	-	62.45 (17.05)	71.40 (13.61)	-	+ 8.90	-
Median score (range)		63 (34–90)	72.5 (45–89)			

**Abbreviations**

MD: Mean Difference

GSE: General Self-Efficacy Scale- 10-item scale: scores range from 10–40

SD: Standard Deviation

LTSES: Leadership/ Teamwork Self-Efficacy Scale- 10-item scale: scores range from 10–50

YLLSDS: Youth Leadership Life Skills Development Survey- 30-item scale: scores range from 0–90.

^1^ No baseline data collected as this survey reflects on gains from the experience being studied.

^2^ The change in mean score between the two time points.

### Thematic analysis

Table 3 presents the three main themes and sub-themes, with illustrative quotes, which were identified from the semi-structured interview data, namely: (1) influence, (2) empowerment, and (3) contribution.

**Table 3:** Themes and sub-themes identified from semi-structured interview data

Theme	Sub-theme	Baseline	Follow-up	Shift* (positive, negative, neutral)
Influence	Representation	*“Young people are very under-represented in society, and everyone should be listened to what they think and be able to express that.”* (M, age 14)	*“there’s a lot of voices in this space of people who are middle aged or older and I really like that this group brings representation to younger voices”* (F, age 17) *“I think the most exciting part of it was to be able to represent them (youth) in like, public articles like the Sydney Morning Herald”* (F, age 16)	Positive
	Voice	*“I think the voices of young people are still not heard enough”* (M, age 16)	*“I wanted, like, to really be able to have my voice heard, and help other people who are struggling with chronic illnesses and diseases. And I feel like that this research project actually did a lot to support that”* (F, age 15)	Positive
	Platform	*“Being part of those groups including these advisory groups gives me safe space to express my opinions and use my voice for what I truly believe in yeah.”* (F, age 16)	*“The group was a really good platform for the issues that I’m interested in, which is youth advocacy and youth health research and obesity research”* (M, age 16)	Neutral
Empowerment	Skill development	*“We need to learn to be discerning and critical in our research for whatever we choose to do in life”* (F, age 17)	*“My skill set has been so broadened by this experience I feel that I probably now would have far more credibility”* (F, age 17)	Positive
	Gaining knowledge	*“I want to learn a lot and just learn more and if in any way give back whether that’s through research or being able to just support research.”* (M, age 17)	*“The confidence and the knowledge I have gained, I feel like it does boost your knowledge and makes you feel like you could lead, and you could do something and take on leadership roles.”* (F, age 15) *“I always feel a little bit more empowered after having these meetings and like I can take action with the knowledge I have gained after the meeting.”* (F, age 17)	Positive
	Opportunities	*“I think I have the potential to make a difference but it’s about whether or not I can access opportunities and avenues where I can actually make change.”* (F, age 17)	*“…having opportunities to speak up about matters that affect me and those of my age.” “Right now, I’m more competent about being able to do things and being able to change stuff.”* (M, age 14)	Positive
Contribution	Meaningful	*“[There’s] not an awareness young people are keen to be involved in these decisions and it’s often just forgotten.”* (F, age 17)	*“You can really strike the comparison between when you are being actively engaged with and active participation is encouraged. And then there’s also research groups that they want to have youth consultants so that they can say they have a youth voice, but they don’t actually want to consult with you. So, I think that’s something that this group does really well, like it is youth focused and youth based and all opinions that are shared are our own, which is really wonderful.”* (F, age 17)	Positive
	Tangible outputs	*“I want to create technology to help people who suffer from chronic illnesses and like diseases as well and all that sort of stuff and make it more accessible within other areas like rural and regional areas as well.”* (F, age 15)	*“With the lancet article that has reached up to thousands of people, I think we’ve definitely been able to make some sort of change in the world as a group.”* (M, age 16)	Positive
	Impact	*“I can have a voice for youth it enables me to have an impact on I guess the broader the wider sort of landscape for youth in the future and allows me to help.”* (M, age 16)	*“With different conferences and being able to talk there and attending the in real life events. Talking to researchers and then yeah, voicing our opinions on Sunrise and in Sydney Morning Herald. Definitely has created an impact.”* (M, age 16)	Positive

**Abbreviations**

F: Female

M: Male

*The shift represents the change of perception status from baseline.

### Influence

Influence was reflected through the desire of participants to have the power to affect change in chronic disease prevention and health research. Within the central theme of influence, three overlapping but separate sub-themes were identified: representation, voice, and platform.

Participants perceived influence as a product of adolescent ‘representation’. Representation was integral to young people perceiving their opinions as valued. Participants also considered adolescent representation necessary for the development of research and interventions that are relevant to young people, to guarantee inclusive research and ensure relatable role models. Participants felt underrepresented in research at baseline, which transformed to feeling represented at follow-up. The evolution of influence through giving young people a voice developed from feeling unheard to being given an active voice in decision-making. Participants associated influence with the need for a ‘platform’ or ‘safe space’ to have their voices heard and influence matters of importance to them. Participants recognised YAGs as an example of an effective ‘platform’ for them to effectively participate in chronic disease prevention research at baseline and this appreciation remained constant over the 12-month study period.

### Empowerment

The theme of empowerment is embodied in the process of the participants gaining confidence especially in controlling their own life and claiming their rights. Three separate but intertwined sub-themes of empowerment were identified: skill development, gaining knowledge and, opportunities. Participants recognised skill development as central to youth empowerment. At baseline skill development was recognised as important for “*study or your career advancement”* with research skills necessary for developing critical thinking skills, at follow-up skill development was also corelated with improved self-efficacy. At baseline participants reported a desire to acquire knowledge about chronic diseases and prevention for the purposes of personal development, to benefit peers, to help with future study and career aspirations, and to learn about research. At follow-up gaining knowledge was associated with increased confidence and self-efficacy. Initially participants felt their ability to make a difference in the chronic disease prevention space was connected to whether they could *“access opportunities”.* At follow-up, participants felt *“more competent”* because of the *“opportunities”* and *“doors that have opened and connections”.*

### Contribution

The desire for participants to be active contributors in health research was evident throughout the study. Contribution as a main theme had three distinct but interconnecting sub-themes: meaningful, tangible outputs, and impact. At baseline participants expressed a lack of ‘meaningful’ contribution on matters concerning them, at follow-up participants reflected positively on their experience of involvement in the YAG and contrasted it to previous experiences in which youth engagement felt like a tokenistic or ‘tick box’ task. Contribution towards ‘tangible outputs’ was another important factor participants valued. At baseline ‘tangible outputs’ were perceived to help improve chronic disease burden and improve access to health promoting resources. At follow-up, tangible outputs such as an essay published in a peer-reviewed journal [3] enabled participants to comprehend the potential of their contribution.

Contribution was significantly underpinned by the desire for participants to make an ‘impact’ on health research related to adolescents. At baseline ‘impact’ was more personal and related to a sense of purpose and being helpful. At follow-up participants perceptions of impact transformed into enabling young people to be impactful in prevention efforts on a personal and community level. Further, recognising that contribution to tangible outputs and opportunities also created impact on research and the broader society *“with different conferences and being able to talk there and attending the in real life events. Talking to researchers and then yeah, voicing our opinions on Sunrise and in Sydney Morning Herald. Definitely has created an impact”.*

### Intersecting themes

**Fig. 2:**
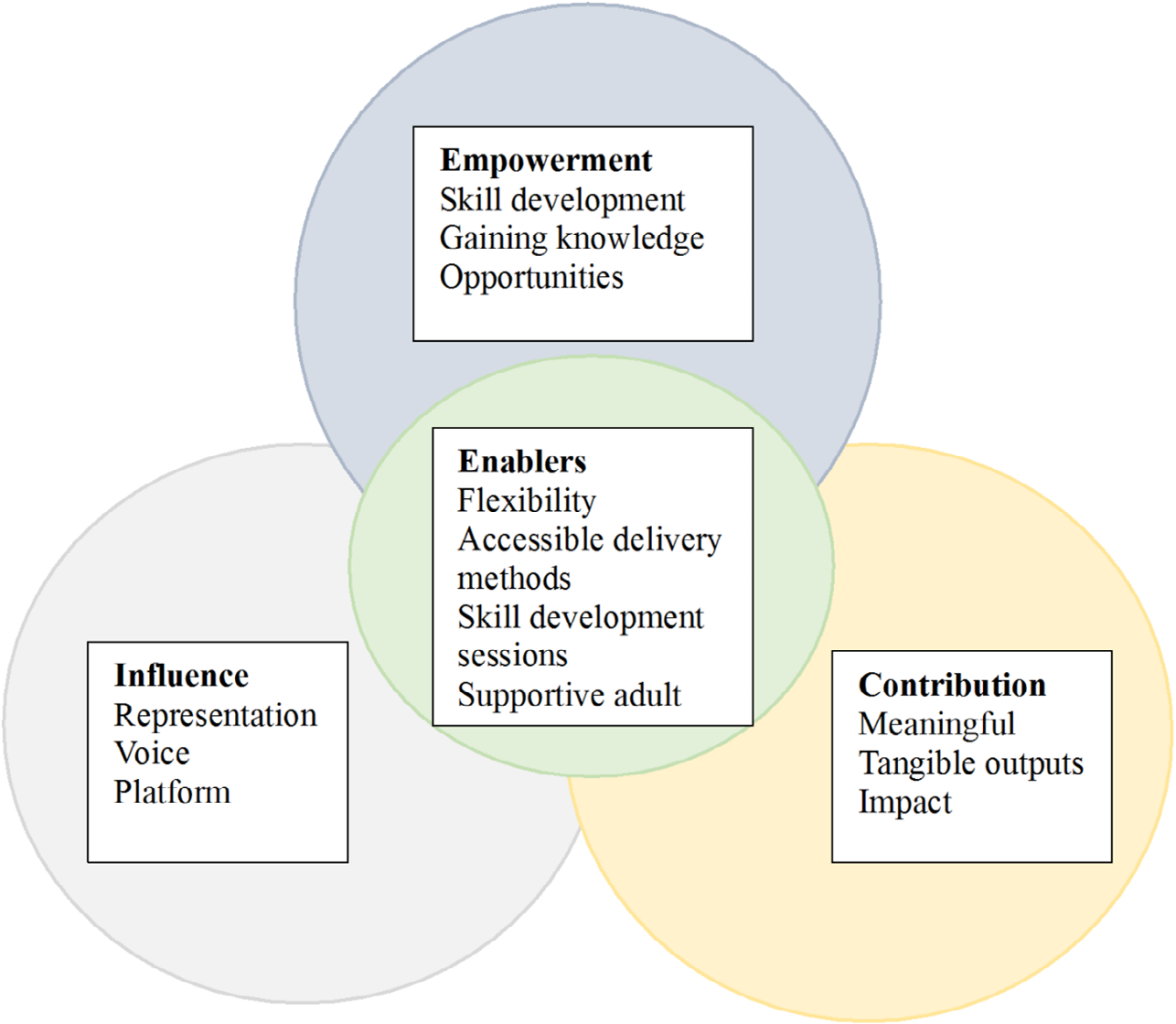
The Intersection between participatory outcomes and process outcomes of a YAG evaluation

Overall, although themes were unique and evolved over the duration of the 12-month YAG term, the YAG framework for collaboration was informed by participatory action research principles which meant that themes and sub-themes were also naturally intertwined (Fig. 2). Participants demonstrated an awareness that learning, knowledge, access to opportunities and capacity to contribute were interlinked to youth empowerment and the ability to have influence over and impact matters of importance to adolescents (Box 1, example 1). Furthermore, participants perceived a platform, meaningful engagement, and tangible outputs to be interrelated with empowerment, the ability to have influence, and impact (Box 1, example 2).

**Box 1:** Supporting quotes for intersecting themes identified from semi-structured interview data

Example 1. Female, age 15: *“When I applied, it was more or less just, I wanted to learn a little bit more about health research, I wanted to contribute to health research, I hadn’t seen any opportunities similar to like, the youth advisory group”, “throughout the programme, our opinion as like younger people was so valued, and we were able to contribute so heavily to it. And it was just really great to know that everyone wanted to know what we thought”, “we have made a bigger difference, and it can be even bigger than that.”*	
Example 2. Male, age 14: *“I feel that there’s a good platform there for any issues that I feel that I [want to] act upon and yes and you feel that as a member of this youth advisory group you can make changes that we can I mean in this article already it’s been it’s been shown that we can if we come together and collaborating with that we can definitely make change and that just translates into the individual people in the YAG going off and also being like inspired that they can make change so yes we can definitely make change.*	

### Process evaluation data

Eight online zoom meetings (mean meeting time 58 min, range 35 to 88; mean attendees 8, range 3–16) and one in-person workshop (7 h total, 9 attendees) were held over the 12-month term. Meetings were held outside of school hours (between 4pm to 7pm) on weekdays (Monday to Friday) or purposely scheduled for school holidays to accommodate the youth advisors’ varying schedules (including, but not limited to, study, family, and casual work commitments). Online polls were conducted on Slack with three options to select a day and time most youth advisors were available. Polls were youth friendly, and members voted using an emoji icon. There were no meetings for three months of the 12-month term due to high school exam periods (November 2021, June 2022) and unplanned youth advisory group events (media engagements regarding youth advisory group’s published essay [3], which required the youth advisors’ time for interviews and photos).

At the first online meeting, all 16 members agreed Slack was the best platform for group activities for the 12-month term and all 16 members joined the Slack workspace when it was established on 18th October 2021. As well, four members of the research team joined as administrators. Of the total 20 members, there were a median of 15 actively weekly members (range 0–19). There was a total of 1,681 messages sent (53% in public channels, 15% in private channels and 32% in direct messages). Administrators sent 789/1681 messages in total (46.9%) and youth advisors sent 892/1681 messages in total (53%; range 1-186 per advisor). Over the 12-months, there was a total of 183 files uploaded for collaborative research or for knowledge sharing. At first, multiple channels were created in the Slack workspace for different research projects and types of conversations. However, members agreed there should be minimal channels for better communication and provided this feedback to the research team. As such, a total of four channels were established, one for general communication and three others for the key research projects the members contributed to over their 12-month term.

### Barriers and enablers to YAG establishment and facilitation

Table 4 presents barriers and enablers to YAG establishment and facilitation. Barriers included time and limited face-to-face components. These barriers were largely due to the nature and complexity of engaging adolescents and COVID-19 restrictions during their 12-month YAG term. Enablers to YAG establishment and facilitation were determined to be flexibility, accessible delivery methods, skill development sessions and a supportive and organised adult facilitator. The barriers and enablers described here are the perception and experience of participants. However, ensuring YAG establishment and facilitation was an iterative process allowed researchers to progressively overcome barriers and strengthen enablers to ensure meaningful adolescent engagement (Fig. 2.).

**Table 4:** Barriers and enablers to YAG establishment and facilitation and lessons learnt

Barriers and enablers	Supporting participant quote	Lessons learnt
Time (barrier)	*“I think another barrier to sort of expressing my opinions with young people’s health and activity have certainly been time I mean like a lot of us are very busy these days and part of that is because we are doing our own health and activities but also there isn’t enough priority given to our wellbeing often it’s forgone in favour of school marks or that sort of thing”* (F, 17)	The research team needs to be flexible with working hours: - Schedule meetings after school hours or in school holidays - Ensure suitability of meeting times by giving youth advisors the opportunity to vote for meeting date and time via a Slack poll.
Limited face-to-face components (barrier)	*“…the lack of face to face, because I struggle with like online stuff, because I get really nervous.”* (F, 15)	Use alternate forms of communication and collaboration to suit a variety of needs e.g., use a platform such as Slack: - Provides channels for discussion on various research projects. - Enables youth advisors to get to know each other better and develop friendships.
Flexibility (enabler)	*“I found the most helpful it’s very flexible like if you just can’t make it that one day or you get to choose like the meeting times on voting if you can’t make it that one day its ok going to be recorded you can still participate and it’s also like it gives you opportunities.“* (F, 15)	The research team needs to be flexible and adaptable to cater to the needs and time of school aged youth. - Meeting times scheduled outside of regular working hours. - Meeting attendance optional, and meeting recordings uploaded to Slack for viewing at youth advisors’ convenience.
Accessible delivery methods (enabler)	*“The use of sort of virtual collaboration so the use of online platform slack and the zoom meetings that we have has really facilitated a lot of discussion both within the meetings but also outside of it, so the groups become quite dynamic and able to accomplish a lot even though we only meet periodically”* (F, 17)	Cater to the needs of geographically distant participants: - Virtual collaboration via multiple methods e.g., Zoom and Slack.
Skill development (enabler)	*“The skill development sessions because they were targeted, they I think they accomplished a lot more than trying to sort of talk about the whole broad world of academic research”* (F, 17)	Ensure meetings enhance participants skills and capacity by incorporating opportunities for: - Gaining knowledge about academic research, and - Skill development
Supportive & organised adult facilitator (enabler)	*“[Researcher name] supporting us through all of it. She really guided us through what it means to be a leader.”* (F, 16) *“Very friendly group. It was led very well. So, it was all well organised.”* (M, 16) *“It’s just really like a really nice environment because everybody is so understanding so kind so nice.”* (F, 15)	A YAG requires a dedicated and organised adult facilitator to: - Build rapport with participants and create a trusting environment. - Support participants to navigate the complexities of academic research. - Connect participants with opportunities and ensure participants are not being tokenised.

**Abbreviations**

M: Male

F: Female

## Discussion

The current evaluation study found that adolescents participating in a YAG perceive adolescent engagement in chronic disease prevention and health research decision-making as essential to both youth capacity building as well as improving youth involvement in chronic disease prevention research. Participation in a YAG led to improvements in indicators of adolescents’ leadership and life skills development. Adolescents reported high general and leadership and teamwork self-efficacy at baseline with no meaningful changes over the YAG term. Further, key outcomes of YAG participation included influence, empowerment, and contribution. Adolescents’ perceived influence to be linked to representation, a youth voice in health advocacy and being provided a platform or safe space to participate. Furthermore, empowerment was perceived to be linked to skill development, gaining knowledge and access to opportunities. Contribution was recognised through adolescents engaging in a meaningful capacity, contributing towards tangible outputs, and making an impact. Overall, our findings indicate that participation in a YAG had a positive effect on adolescents’ skill development as well as perceptions related to research and chronic disease prevention. Process data revealed synergies between process indicators which enable YAG facilitation and enhanced participatory outcomes, indicating that a YAG is a feasible method to meaningfully engage adolescents in all stages of research processes.

Overall, there is limited evidence of the impact of participation in a YAG on adolescent’s or chronic disease prevention research [32]. Nonetheless, our study results are consistent with findings from the broader YPAR literature. A systematic review of 63 YPAR studies in the United States found the most common outcomes developed by youth to be those related to agency and leadership followed by academic or career outcomes [30]. Furthermore, another systematic scoping review found meaningful collaboration between researchers and youth enabled adolescents involved in participatory research the ability to influence research and development processes [32]. In partnership with the research team, an outcome of our YAG was a published an essay in a peer-reviewed journal [3]. This example of a tangible output of adolescent participation from a YAG that enabled adolescents to highlight their perspective on youth health prioritise and served as a foundational element in the research teams youth heath research agenda [3]. A recent systematic review and meta-analysis of methods and effects of engaging relevant consumers in research determined the enhanced relevance and positive outcomes for health research resulting from youth engagement [17]. Similarly, global consultations with young people have confirmed this message, youth calling for transgenerational equal partnerships with young people and influence over agenda setting as a crucial step to securing adolescent wellbeing for the future [27, 28, 61].

Our results fill a gap in the chronic disease prevention literature, demonstrating the potential adolescent engagement provides for enhancing research practice but also upskilling and empowering adolescents [30]. Improvements in YAG members perceived skill development and capacity building was an expected outcome of YAG participation. Meaningful and effective adolescent engagement is often inhibited by difficulties including the need to prepare and upskill youth for engagement activities [40, 62]. To overcome this issue and enhance participatory research quality, the research team integrated skill development sessions into the regular YAG monthly meetings. Skill development sessions were reported by adolescents to be a key enabler to ongoing participation in the YAG. A scoping review of youth engagement in chronic disease prevention literature [31, 32] determined that participatory activities have the potential to improve capacity building, empowerment, influence, and confidence. It was also evident that increasing adolescent involvement in the form of youth advisory groups, co-design and decision-making processes contributed to more meaningful obesity and chronic disease prevention associated outcomes. Meaningful participation led to improvements in empowerment and influence [63, 64] of the participating youth [65] and their peers [66–68].

The YAG provoked unanticipated interest across multiple sectors including academia, public, media and government sectors. Interest in the YAG’s youth-led health research provided further engagement and advocacy opportunities for adolescents and enhanced their reach and influence. The YAG gave adolescents a platform to raise awareness and appreciation of the need for youth representation and a voice in the chronic disease prevention research agenda. A systematic review assessing YPAR in youth substance use prevention research found that adolescent participation increased community awareness of the problem and potential solutions [69]. Furthermore, adolescent engagement supported change by generating youth specific research, data and prevention materials [69].

The process of establishing and facilitating a YAG focused on health research required a supportive, iterative, and flexible approach to ensure adolescents remained engaged in a fulfilling and meaningful capacity. Similarly, although in a mental health context, a critical and reflective attitude to overcoming barriers was found to be pivotal to increasing youth engagement and enhancing youth related research outcomes in the Matilda Centre Youth Advisory Board [40] and the Oxford Neuroscience, Ethics and Society Young People’s Advisory Group (NeurOx YPAG) [70]. The CO-CREATE project for obesity prevention research and policy action also identified the need for ethical considerations when engaging youth [71]. Still, the large number of participants and logistics of a multi-country study amplified barriers to engaging adolescents [71]. Similarly to our YAG, the CO-CREATE project highlights the requirement of a flexible and youth specific framework to ensure the process of adolescent participation is safe and empowering [72].

Our study demonstrated that a YAG exemplifies a novel youth engagement method which provides adolescents the opportunity to influence all stages of the research and development process. Advisory groups with adult participants have demonstrated improvement in health research and knowledge translation for healthy public policy [73]. Similarly, the literature indicates that meaningful adolescent engagement based on YPAR principles has the potential to generate individual capacity building and health benefits for participating youth [23, 30, 61, 74]. On a broader scale, participatory research has been associated with improvements in health outcomes by ensuring research is relevant and acceptable to those it is intended for [75]. Adolescent engagement enhances research agendas and methodology by ensuring research conduct is targeted and specific to youth [30, 43]. Evidence from a Canadian YAG focused on child participatory research found that youth involvement adds a critical ethical element to research methods [76]. Moreover, youth advisory structures with a specific research focus such as health services research in pharmacy [75] and overweight and obesity prevention [77] have been shown to add value to enhancing research and implementing youth centred solutions. Furthermore, meaningful youth engagement and co-design of research via participation in a YAG, has the potential to improve distribution of medical research funding [78]. Directing chronic disease prevention research funding towards research priorities and interventions which are targeted, and identified by the target population as relevant and acceptable is key to improving population based health solutions [79].

### Limitations

We endeavoured to conduct the study as rigorously as possible however there are some limitations to the study which are important to note. Our research study was predominantly based on qualitative research methods. Innate limitations of qualitative research [80] include that the quality of the research is reliant on the specific skills of the researcher and therefore more easily influenced by the researcher’s individual subjective biases and interests. Participant responses may have been influenced by the presence of the researcher during data collection methods such as the semi-structured interviews.

Additional limitations specific to the development and facilitation of the YAG should be noted. Most of the adolescents who applied to participate in the YAG either had previous leadership experience and were keen to have a career in the research or health sectors. Further, participants recruited to participate in the YAG were chosen through a selection process in which participants who had more research or leadership experience or demonstrated potential were more likely to be selected. Survey and interview data conducted at baseline evidenced this via high baseline self-efficacy, leadership, and teamwork skills. It is apparent that adolescents with high baseline self-efficacy and leadership skills are more likely to apply for such opportunities, however it is important to identify how to engage a diverse sample of adolescents in chronic disease prevention research. Future YAGs may consider baseline assessments of self-efficacy to ensure adolescents with less belief in their general capacities and leadership and teamwork capacities can participate and develop their skills. Furthermore, only 16 adolescents were recruited to participate in the YAG. This small sample size was important as it enabled participants to build rapport with the research team, while a larger group may have increased feelings of tokensim and made meaningful adolescent engagement difficult [70]. Finally, the projects the YAG participants contributed to are ongoing, therefore it is difficult to evaluate the effect of their participation on research outcomes.

## Conclusion

Meaningful youth engagement in a YAG based on YPAR principles generated numerous benefits for adolescents personally and for chronic disease prevention research more broadly. Improvements in participating adolescents’ leadership skills, together with enhanced participatory markers of empowerment and influence enabled adolescents to advocate for and contribute towards chronic disease prevention research for young people. Enhanced participatory outcomes were linked to successful facilitation of a YAG, which required the research team to be iterative, flexible, and adaptable in their approach, thus strengthening enablers and overcoming barriers to establishment and facilitation of the YAG.

## Data Availability

All data generated or analysed during this study are included in this published article.

## List of abbreviations

YAG: Youth Advisory Group
YPAR: Youth Participatory Action Research
UNICEF: United Nations Children’s Fund
COVID-19: Corona Virus Disease 2019
UNCRC: United Nations Convention on the Rights of a Child
WHO: World Health Organization
SDG: Sustainable Development Goal
AUD: Australian Dollar
SEIFA: Socioeconomic Indexes for Areas
IRSD: Index of Relative Socioeconomic Disadvantage
ASGS-RA: Australian Statistical Geographical Standard Remoteness Areas
GSE: General Self-Efficacy scale
LTSES: Leadership/ Teamwork Self-Efficacy Scale
YLLSDS: Youth Leadership Life Skills Development Survey
REDCap: Research Electronic Data Capture
SD: Standard Deviation
NeurOx YPAG: Oxford Neuroscience, Ethics and Society Young People’s Advisory Group
